# Blood Culture Contamination Creep Independent of COVID-19 Pandemics: An Interrupted Time-Series Analysis

**DOI:** 10.3390/antibiotics14060533

**Published:** 2025-05-22

**Authors:** Samo Jeverica, Jani Dernič, Peter Golob, Alenka Stepišnik, Bojan Novak, Tomaž Gantar, Lea Papst, Anamarija Juriševič Dodič, Darja Barlič Maganja, Jan Zmazek, Mladen Gasparini

**Affiliations:** 1 Izola General Hospital, 6310 Izola, Slovenia; jani.dernic@sb-izola.si (J.D.); peter.golob@sb-izola.si (P.G.); alenka.stepisnik@sb-izola.si (A.S.); bojan.novak@sb-izola.si (B.N.); tomaz.gantar@sb-izola.si (T.G.); mladen.gasparini@sb-izola.si (M.G.); 2 Faculty of Health Sciences, University of Primorska, 6000 Koper, Slovenia; darja.maganja@fvz.upr.si; 3 Department of Infectious Diseases, University Medical Centre Ljubljana, 1000 Ljubljana, Slovenia; lea.papst@kclj.si; 4 Department of Medical Microbiology Koper, National Laboratory of Health, Environment and Food, 2000 Maribor, Slovenia; anamarija.jurisevic.dodic@nlzoh.si; 5 Faculty of Natural Sciences and Mathematics, University of Maribor, 2000 Maribor, Slovenia; jan.zmazek@um.si

**Keywords:** blood culture, blood culture contamination, COVID-19, Slovenia, interrupted time-series analysis

## Abstract

**Background/Objectives**: Our study aimed to assess longitudinal trends in blood culture contamination in a regional secondary care teaching hospital before and after the COVID-19 pandemic and to evaluate differences in the interpretation of trends using two distinct quasi-experimental statistical methods, including interrupted time-series analysis. **Methods**: We analyzed data from a 10-year period spanning from 2015 to 2024, encompassing 147,733 admissions and 634,158 patient-days, as well as a total of 25,068 blood cultures. The (i) blood culture contamination rate, (ii) contaminant proportion, (iii) single blood culture rate, and (iv) first-to-second bottle contamination ratio were calculated. **Results**: The observed usage rate of blood cultures per 1000 patient-days was 38.9. The contamination rate of blood cultures increased from 0.9% to 1.5% (*p* = 0.001) in the post-COVID-19 period, accompanied by a rise in the proportion of contaminant bacteria from 9.8% to 14.2% (*p* = 0.016). Additionally, the proportion of single blood culture collections increased from 23.1% to 33.6% (*p* < 0.001). Finally, the overall first-to-second bottle contamination ratio was 1.54, while the ratio in the post-COVID-19 period was 1.92. **Conclusions**: In a low-COVID-19-burden secondary care teaching hospital setting, blood culture contamination rates have progressively increased over the past decade, irrespective of the pandemic. These findings underscore the importance of sustained vigilance in infection prevention and control practices, strict adherence to blood culture collection protocols, and the ongoing need for staff training.

## 1. Introduction

Sepsis is a life-threatening condition resulting from a dysregulated host response to infection and represents a significant global health burden. In 2017, an estimated 48.9 million cases occurred worldwide, leading to 11.0 million deaths that accounted for nearly 20% of all global mortality [1]. Blood cultures remain one of the most critical diagnostic tools in clinical microbiology and are considered among the most sensitive culture techniques, largely due to the optimized composition of culture media and the use of automated systems for continuous growth detection [2,3]. However, bacteremia may also occur under non-pathological conditions, during localized infections, or as a result of contamination introduced during sample collection. These factors can compromise the specificity of positive blood culture results [4,5,6]. Despite their importance, the majority of blood cultures performed in modern microbiological laboratories yield negative results [4,6]. This can be attributed to a poor selection of patients for blood cultures, primarily those with conditions that rarely result in detectable bacteremia, such as patients with isolated fever, leukocytosis, or uncomplicated infections like community-acquired pneumonia, skin and soft tissue infections, or urinary tract infections with systemic symptoms. Additionally, technical factors such as the collection of insufficient blood volume or a single blood culture set, instead of multiple sets, further diminish the sensitivity and clinical utility of blood cultures [4,7].

Blood culture contamination imposes a substantial burden on patient care by prolonging hospital stays, leading to unnecessary antibiotic therapy, escalating diagnostic and therapeutic interventions, and increasing overall healthcare costs [2,8,9]. Current performance benchmarks recommend maintaining contamination rates below 3%. However, recent evidence suggests that considerably lower contamination rates are attainable, supporting a shift toward the zero-contamination goal [8]. To that end, the American Society for Microbiology has published evidence-based practice guidelines focused on contamination reduction strategies [10]. Two types of metrics can be used to describe blood culture contamination magnitude: (i) the percentage of all blood cultures that are contaminated and (ii) the percentage of positive blood cultures that yield organisms classified as contaminants. The main difference between them lies in the denominator, which is much smaller when counting only positive samples [2,8].

Since 2019, the COVID-19 pandemic has resulted in over 7 million reported deaths, according to the World Health Organization. However, the actual excess mortality was estimated to be 2.74 times higher [11]. Slovenia detected its first COVID-19 case in March 2020 and declared an epidemic shortly thereafter. The first wave was relatively mild, never exceeding 61 positive molecular tests per day. The second wave began in September 2020 and lasted until July 2021, when the Omicron variant triggered the third wave, which persisted until 2022. As of March 2023, Slovenia has reported 9201 COVID-19 related deaths [12,13]. The pandemic presented significant challenges to healthcare systems globally, including shortages of medical personnel, regular and intensive care beds dedicated to COVID-19 patients, and personal protective equipment (PPE). The hospital environment became increasingly complex due to the extensive utilization of PPE, making adherence to standard infection prevention and control principles difficult [14,15]. Blood cultures are an essential diagnostic tool in the management of critically ill patients. During the pandemic, several changes were reported in both the utilization and diagnostic yield of this method, some of which have persisted in the post-pandemic period [15,16,17,18,19,20].

This study aimed to assess longitudinal trends in blood culture contamination at a regional secondary care teaching hospital before and after the onset of the COVID-19 pandemic, as well as to evaluate how trends interpretation varies using two distinct quasi-experimental statistical methods, including interrupted time-series analysis.

## 2. Results

We analyzed data from a 10-year period from 2015 to 2024, encompassing 147,733 admissions and 634,158 patient-days. Within this interval, a total of 25,068 blood cultures were collected, representing a usage rate of 38.9 blood cultures per 1000 patient-days. No statistically significant differences were observed in the frequency of blood culture usage overall, or specifically among adult and pediatric patients, between the pre- and post-COVID-19 periods (Table 1). Among all the blood cultures obtained, 303 (1.2%) were classified as contaminated. The median age of patients with blood culture contamination was 66 years (interquartile range [IQR]: 49–76); of these patients, 64.7% were male, and 85.5% were categorized as adults (Table 2).

The majority of blood cultures were obtained from the Department of Internal Medicine (58.4%), followed by the Department of Surgery (18.8%) and the Emergency Department (13.2%). Coagulase-negative staphylococci were the most frequently identified contaminants, accounting for 64.4% of cases, followed by streptococci (13.2%) and *Cutibacterium* spp. (6.3%) (Table 2). A small proportion of contaminations were polymicrobial in nature (6.6%). Contaminated blood cultures were most commonly collected via percutaneous venipuncture (54.5%). However, during the post-COVID-19 period, a higher proportion of contaminated samples was obtained through intravascular catheters (51.7%) (Table 2).

**Figure 1:**
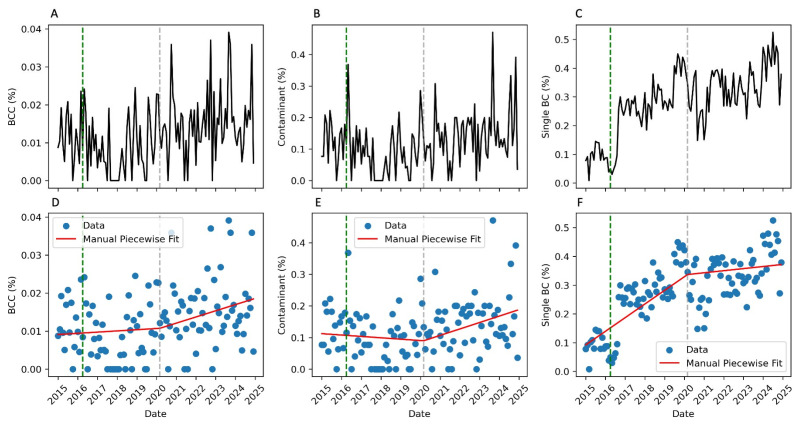
Interrupted time-series analysis with segmented linear regression plots (Manual Piecewise Fit) for the blood culture contamination (BCC) rate (**A**,**D**), the contaminant proportion (**B**,**E**), and the single blood culture (BC) rate (**C**,**F**). The grey vertical line represents the changepoint between the pre- and post-COVID-19 periods. The green vertical line represents the opening of the new Emergency Department.

Blood culture contamination indicators for the entire study period, as well as for the pre- and post-COVID-19 periods, are summarized in Table 3. A significant increase in blood culture contaminations was observed, rising from 0.9% in the pre-pandemic period to 1.5% (*p* = 0.001) in the post-pandemic period. This was accompanied by a rise in the proportion of contaminant bacteria among all positive blood cultures, rising from 9.8% to 14.2% (*p* = 0.016). This upward trend was temporally associated with a significant increase in the frequency of single blood culture collections, which rose from 23.1% to 33.6% in the post-COVID-19 period (*p* < 0.001). Notably, a marked increase in the proportion of single blood cultures was already observed in 2016, coinciding with the opening of the novel Emergency Department at the IGH, a period that also saw a rise in emergency visits (Figure 1C and Figure S1).

Nevertheless, an interrupted time-series analysis employing the segmented linear regression model demonstrated a substantially superior fit compared to the conventional linear regression model, albeit exclusively for the single BC rate indicator. Conversely, there was no significant difference observed for the BCC rate and contaminant proportion (Table 4). These findings suggest a gradual upward trend in the occurrence of contamination events over time, rather than an abrupt change directly attributable to the COVID-19 pandemic (Figure 1A–F).

Finally, after excluding anaerobic contaminants (due to the preferential growth of anaerobic microorganisms in anaerobic bottles, which are typically collected second) and excluding contaminants from pediatric blood cultures (where only a single bottle is collected), the analysis of the first-to-second bottle contamination ratio revealed a consistently higher contamination rate in the first bottle. The overall ratio was 1.54, increasing to 1.92 during the post-COVID-19 study period (Table 3).

## 3. Discussion

This study provides a comprehensive analysis of blood culture contamination trends over a 10-year period in a regional secondary care teaching hospital, with a specific focus on the impact of the COVID-19 pandemic. We detected a significant increase in blood culture contamination in the post-COVID-19 period (from 0.9% to 1.5%); however, this increase was gradual in nature and, therefore, not directly attributable to the pandemic as the segmented linear regression model fit did not exhibit a statistically significant difference between the pre- and post-COVID-19 periods regarding the blood culture contamination rate and the proportion of contaminants. Coagulase-negative staphylococci were the predominant contaminants identified. Additionally, we observed a relatively high rate of single blood culture collections, which coincided with the opening of the new Emergency Department. Finally, our data demonstrate that the first blood culture bottle of a set is significantly more likely to be contaminated, with the contamination ratio reaching 1.92 in the post-COVID-19 period. This finding underscores the need for stricter collection site preparation protocols or the implementation of initial sample diversion techniques to reduce contamination rates.

The association between the COVID-19 pandemic and increased rates of blood culture contamination has been examined in several studies, with most reporting an elevation in contamination rates attributable to various factors. Specifically, Andrei et al. observed a reduction in blood culture utilization accompanied by increased contamination rates during the pandemic period compared to pre-pandemic data (2020 vs. 2016) in a COVID-19 dedicated tertiary care hospital in Romania [20]. Similarly, Saleh et al. presented longitudinal data from a tertiary care medical center in Lebanon, indicating a notable increase in blood culture contamination rates during the 2020–2021 pandemic period, followed by a subsequent decline in 2022 [21]. Additionally, Farfour et al. analyzed data from two French hospital microbiological laboratories across the first and second pandemic waves, reporting elevated blood culture utilization and contamination rates during the initial wave; however, these increases were not observed during the subsequent wave [18]. Conversely, Tolle et al. reported a non-reversible, sustained increase in blood culture contamination rates that persisted even after the peak of the pandemic [17]. Sacchetti et al. identified several key contributors to blood culture contamination in a tertiary care center in the United States, including an increased number of blood cultures collected per month and a larger number of beds per unit [19]. Furthermore, Park et al. concluded that the primary driver of increased contamination was the use of complex PPE in COVID-19 wards, suggesting that simplifying PPE could improve adherence to aseptic techniques and thereby reduce contamination rates [15]. In our study, we observed a marked increase in blood culture contamination rates following the onset of the COVID-19 pandemic. However, this rise did not occur at a single identifiable time point but instead evolved gradually over an extended period. This trend likely reflects a complex and multifactorial process, potentially involving a progressive decline in adherence to aseptic techniques, staffing shortages, increased workload, suboptimal working conditions, and a higher proportion of blood cultures collected via indwelling catheters. Patient-related factors may have also contributed to this ongoing increase, although this was not assessed in our study.

Blood culture usage rates vary significantly across hospitals, wards, and specialties. Given that the majority of blood cultures yield negative results, even in settings with low contamination rates, a substantial proportion of blood culture isolates may still represent contaminants. Recent ESCMID guidelines on antimicrobial stewardship in Emergency Departments recommend against the routine collection of blood cultures for common conditions such as community-acquired pneumonia, urinary tract infections with systemic symptoms, and uncomplicated skin and soft tissue infections in the absence of sepsis [5]. Currently, no national or international benchmarks exist for optimal blood culture utilization. However, a recent retrospective study conducted in the United States revealed mean utilization rates of 273 and 146 per 1000 patient-days in medical and surgical intensive care units (ICUs), respectively [16]. Additionally, the study reported blood culture utilization rates of 65–80 per 1000 patient-days in medical and surgical wards. Single blood culture episodes accounted for less than 10% in 97% of units, while the blood culture contamination rates remained below 1% in 51% of units. Using regression modeling techniques, the study proposed minimum utilization thresholds of 120, 80, and 30 blood cultures per 1000 patient-days for medical ICUs, medical–surgical ICUs, and medical–surgical wards, respectively [16]. Utilization rates below these values may indicate the underutilization of this important diagnostic tool. In comparison, our findings indicate suboptimal adherence to the standard recommendation of collecting at least two blood culture sets per episode, which may negatively impact diagnostic accuracy and clinical decision-making. Over a quarter of all blood cultures, and more than one-third of those collected since the onset of the COVID-19 pandemic, were obtained as single sets. Moreover, based on the proposed benchmark thresholds, overall blood culture utilization at IGH may fall near the lower acceptable limit. This is particularly noteworthy considering that IGH includes both medical and surgical ICUs, where more frequent blood culture collection would typically be expected due to the higher acuity of patients. Nevertheless, alternative benchmarks may be more suitable for Slovenian secondary care hospitals.

Among the relatively novel and infrequently implemented strategies for reducing blood culture contamination is the use of initial specimen diversion devices or techniques. These devices function by diverting a small volume of blood (typically between 0.15 mL and 2 mL) that may contain skin flora or dermal debris introduced during venipuncture. Such contaminants are not always fully eliminated by standard skin antisepsis procedures [2]. Currently, at least two commercially available devices are specifically designed to mitigate blood culture contamination: Steripath (Magnolia Medical Technologies, Seattle, WA, USA) [22,23] and Kurin (Kurin Inc, San Diego, CA, USA) [24]. In addition to these devices, simpler techniques have also been proposed to reduce contamination, using an initial blood collection tube to divert the first few milliliters of blood [25] or modifying the order of drawing by collecting blood for biochemical analysis before blood cultures [26]. At our institution, none of these alternative techniques are currently implemented. On the contrary, since 2011, a strict protocol has mandated that blood cultures be collected before any other blood samples, with the aerobic bottle drawn prior to the anaerobic bottle. Furthermore, the protocol for blood culture collection remained consistent throughout the entire study period. After controlling for all potential confounding factors, we observed a nearly twofold increase in contamination in the first bottle compared to the second bottle when a full set of bottles was collected. A recent systematic review and meta-analysis demonstrated that the use of initial specimen diversion was associated with a substantial reduction in contamination, with an odds ratio of 0.26 [23]. Moreover, the American Society for Microbiology has recently recommended the use of initial specimen diversion devices, citing research that reported a 64% reduction in blood culture contamination rates [10]. Our results support this concept, although our data suggest a somewhat more modest potential for contamination reduction.

Our study has several limitations. First, contaminants were categorized based on microbiological criteria, excluding isolates not listed among the CDC-defined common commensals, in order to ensure standardization and comparability. This exclusion was made because commensals originating from mucosal surfaces may indicate asymptomatic bacteremia rather than contamination introduced during skin antisepsis. Second, the retrospective design of this study did not allow for the identification of which specific factors contributed most significantly to the observed increase in blood culture contamination over time. Thirdly, although the protocol for both blood culture collection and the laboratory processing of blood cultures remained unchanged, there may still have been some minor differences that were dependent on staff training and protocol compliance. More positively, our dataset spans a ten-year period and represents the first benchmarking effort at our institution, providing a solid foundation for future interventional studies aimed at eliminating blood culture contamination from our clinical setting.

## 4. Materials and Methods

### 4.1. Study Design and Clinical Setting

This was an observational retrospective cohort study conducted at Izola General Hospital (IGH), a regional secondary care teaching hospital located in the Coastal–Karst region of Slovenia, near the borders with Italy and Croatia. IGH serves a population of approximately 170,000 and has a capacity of 300 acute care beds. Each year, the hospital records around 15,000 patient admissions and approximately 63,000 patient-days. The hospital is organized into five main departments: internal medicine, surgery, pediatrics, gynecology, and emergency medicine. It also includes two intensive care units located within the internal medicine and surgery departments.

During the COVID-19 pandemic, the hospital functioned as a mixed-type facility, providing care for both COVID-19 and non-COVID-19 patients. In 2020, 2021, and 2022, COVID-19 cases accounted for 5.2%, 9.5%, and 5.1% of total patient-days, respectively, indicating a relatively low burden of COVID-19 hospitalizations [13]. The study period spanned from 1 January 2015 to 31 December 2024. For the purposes of analysis, the timeframe was divided into two periods: the pre-COVID-19 period (1 January 2015 to 31 March 2020) and the post-COVID-19 period (1 April 2020 to 31 December 2024).

### 4.2. Blood Culture Collection

Although the hospital does not have a dedicated phlebotomy team, blood culture collection is a standardized procedure performed by senior ward nurses. A formal collection protocol has been in place since 2011 and includes several key elements to ensure aseptic technique use and minimize contamination. These measures include the use of PPE (at minimum surgical masks, head covering and sterile gloves), a dedicated sterile blood culture collection set for skin preparation, and a skin antiseptic solution containing 70% isopropyl alcohol and 0.5% chlorhexidine. Skin antisepsis was performed using a triple-application technique, allowing a minimum of 30 s between applications to ensure adequate contact time. A single blood draw (i.e., one blood culture) consisted of two blood culture bottles (aerobic and anaerobic) in adult patients and one pediatric blood culture bottle in pediatric patients. According to the protocol, blood cultures were collected prior to any biochemical sampling, with aerobic bottles inoculated before anaerobic ones. Additionally, as part of routine practice, the tops of the blood culture bottles were disinfected with alcohol pads prior to inoculation.

### 4.3. Microbiological Methods, Definitions, and Indicators

Throughout the study period, the BD BACTEC FX automated blood culture system was used, utilizing Standard Aerobic, Lytic Anaerobic, and Peds Plus/F bottles for adult aerobic, anaerobic, and pediatric blood cultures, respectively. Positive blood culture bottles were subcultured on appropriate agar media, and microbial identification was performed using the MALDI Biotyper system (Bruker Daltonics, Bremen, Germany). Antimicrobial susceptibility testing was conducted using the disk diffusion method, in accordance with the most recent guidelines provided by the European Committee on Antimicrobial Susceptibility Testing (EUCAST). Negative culture bottles were incubated for a total of five days before being reported as negative.

Isolates were stratified by bottle type (aerobic, anaerobic, pediatric), collection order (first, second), and collection method (percutaneous, catheter). They were then categorized as either contaminants or non-contaminants based on established blood culture contamination criteria. A blood culture was considered contaminated if a typical skin commensal organism was isolated from a single collection (one or two bottles) within a 24 h period, regardless of the total number of sets obtained [2,3,9]. Organisms classified as typical skin commensals included coagulase-negative staphylococci, *Bacillus* spp., *Cutibacterium* spp., *Corynebacterium* spp., and *Micrococcus* spp. The updated list of common commensals from the Centers for Disease Control and Prevention and the National Healthcare Safety Network (NHSN) was used as a reference [27].

Four indicators were used to assess blood culture contamination. First, the blood culture contamination rate was defined as the proportion of all collected blood cultures yielding a contaminant microorganism. Second, the contaminant proportion was defined as the proportion of contaminants among all unique blood culture isolates. Third, the single blood culture rate represented the proportion of patients from whom only one blood culture set was collected. Finally, the first-to-second bottle contamination ratio was calculated by dividing the number of contaminants isolated from the aerobic (first) bottle by the number of those isolated from the anaerobic (second) bottle. For this analysis, contaminants from pediatric bottles (which are not collected in pairs) and obligate anaerobes (which predominantly grow in anaerobic bottles) were excluded.

### 4.4. Statistical Methods

Two types of before-and-after analyses were conducted using JASP (version 0.19.0; University of Amsterdam, Amsterdam, The Netherlands). First, a Mann–Whitney U-test was employed to compare monthly contamination rates between the pre- and post-COVID-19 periods. Second, the first-to-second bottle contamination ratio was compared using a chi-square test for independent proportions. Finally, an interrupted time-series analysis was performed using the segmented linear regression model employing the pwlf Python library (Piecewise Linear Fit, version 2.5.1) [28]. The change point was set to 1 April 2020. Statistical significance was corrected for multiple comparisons using Bonferroni correction (*n* = 3) across comparisons and defined as *p* < 0.05.

## 5. Conclusions

In a hospital setting with a low incidence of COVID-19-related admissions, we observed a gradual increase in blood culture contamination rates over the past decade. Importantly, this trend was not significantly exacerbated by the COVID-19 pandemic. Our findings highlight the critical need for sustained efforts in infection prevention and control practices, along with strict adherence to standardized blood culture collection protocols, ongoing staff training, and regular performance monitoring. Additionally, the implementation of supplementary methods such as initial specimen diversion techniques may enable further reductions in contamination rates.

## Figures and Tables

**Table 1:** Hospital level characteristics.

Characteristic	Pre-COVID-19		Post-COVID-19		All		*p*-Value
	*n*	(%)	*n*	(%)	*n*	(%)	
Hospital							
Admissions sum	79,608	(53.9)	68,125	(46.1)	147,733	(100)	
Patient-day sum	347,504	(54.8)	286,654	(45.2)	634,158	(100)	
Patient-day median (IQR) ^a^	5447	(5115–5881)	5013	(4610–5441)	5267	(4874–5653)	<0.001
Blood culture (BC)							
BC sum	13,968	(55.7)	11,100	(44.3)	25,068	(100)	
BC/PD ^b^ median (IQR) ^a^	39.6	(35.6–44.8)	37.8	(34.8–43.2)	38.9	(35.1–44.1)	0.161
Adult BC/PD ^b^ median (IQR) ^a^	35.0	(31.3–40.3)	34.1	(30.1–38.7)	34.7	(30.8–39.9)	0.825
Pediatric BC/PD ^b^ median (IQR) ^a^	4.3	(3.45–5.2)	4.3	(3.4–5.4)	4.3	(3.4–5.3)	0.173

^a^ interquartile range; ^b^ blood cultures per 1000 patient-day monthly intervals.

**Table 2:** Blood culture contamination characteristics.

Characteristic	Pre-COVID-19		Post-COVID-19		All		*p*-Value
	*n*	(%)	*n*	(%)	*n*	(%)	
Demographic							
BCC ^$^	131	(43.2)	172	(56.8)	303	(100)	
Male	80	(61.1)	116	(67.4)	196	(64.7)	0.222
Female	51	(38.9)	55	(32.6)	106	(35.3)	
Age median (IQR) ^a^	66	(45–77)	66	(49–76)	66	(49–76)	0.820
Patient group							
Adult	112	(85.5)	147	(85.5)	259	(85.5)	0.994
Pediatric	19	(14.5)	25	(14.5)	44	(14.5)	
Department							
Internal medicine	82	(62.6)	95	(55.2)	177	(58.4)	0.001
Surgery	26	(19.8)	31	(18.0)	57	(18.8)	
Pediatrics	16	(12.2)	11	(6.4)	27	(8.9)	
Emergency	4	(3.1)	33	(19.2)	37	(13.2)	
Gynecology	3	(2.3)	2	(1.2)	5	(1.7)	
Microorganism groups							
CoNS *	76	(58.0)	119	(69.2)	195	(64.4)	0.193
Streptococcus	23	(17.6)	17	(9.9)	40	(13.2)	
Cutibacterium	8	(6.1)	11	(6.4)	19	(6.3)	
Corynebacterium	7	(5.3)	4	(2.3)	11	(3.6)	
Anaerobes	4	(3.1)	4	(2.3)	8	(2.6)	
Bacillus	3	(2.3)	1	(0.6)	4	(1.3)	
Other	10	(7.6)	16	(9.3)	26	(8.6)	
Polymicrobial BCC ^$^							
Yes	6	(4.6)	14	(8.1)	20	(6.6)	0.216
No	125	(95.4)	158	(91.9)	283	(93.4)	
Collection type							
Percutaneous	82	(62.6)	83	(48.3)	165	(54.5)	0.013
Catheter	49	(37.4)	89	(51.7)	138	(45.5)	

^$^ blood culture contamination; ^a^ interquartile range; * CoNS: coagulase negative staphylococci.

**Table 3:** Blood culture contamination indicators.

BCC ^#^ Indicators	Pre-COVID-19		Post-COVID-19		Total		*p*-Value ^b^
	%	(95% CI)	%	(95% CI)	%	(95% CI)	
BCC ^#^ rate	0.9	(0.8–1.1)	1.5	(1.3–1.8)	1.2	(1.1–1.4)	0.001
Contaminant proportion	9.8	(7.8–11.8)	14.2	(11.8–16.6)	11.9	(10.3–13.5)	0.016
Single BC * rate	23.1	(20.2–26.1)	33.6	(31.5–35.8)	28.1	(26.0–30.2)	<0.001
First-to-second bottle ratio (n1, n2, all) ^a^	0.886	(31, 35, 66)	1.917	(115, 60, 175)	1.537	(146, 95, 241)	0.024

^#^ blood culture contamination; * blood culture; ^a^ n1 first bottle; n2 second bottle; all first and second bottle; ^b^ Bonferroni correction (*n* = 3) was applied.

**Table 4:** Interrupted time-series parameters, slope coefficient, and segmented linear regression model fit improvement significance (*p*-value).

Interrupted Time Series Parameters	Pre-COVID-19	Post-COVID-19	*p*-Value
	Slope [×10^−3^]	Slope [×10^−3^]	
BCC ^#^ rate	0.350	1.620	0.700
Contaminant proportion	−3.960	20.591	0.065
Single BC * rate	46.187	7.474	<0.001

^#^ blood culture contamination; * blood culture.

## Data Availability

Data are contained within this article.

## Supplementary Materials

The following supporting information can be downloaded at: https://www.mdpi.com/article/10.3390/antibiotics14060533/s1, Figure S1: Number of Emergency department visits during the study period. The opening of the department dates to the begining of 2026.

## Abbreviations

The following abbreviations are used in this manuscript:

IGH	Izola Genera Hospital
BC	Blood culture
BCC	Blood culture contamination
ICU	Intensive care unit
IQR	Interquartile range
PD	Patient-day
