# Supplementary material for: Blood Culture Contamination Creep Independent of COVID-19 Pandemics: An Interrupted Time-Series Analysis
Source: Antibiotics (Basel). 2025 May 22;14(6):533. doi: 10.3390/antibiotics14060533 (PMC12189594; doi:10.3390/antibiotics14060533)
Supplement: Supplementary file 1 [file antibiotics-14-00533-s001.zip › antibiotics-3632368-supplementary.pdf]

Supplementary material

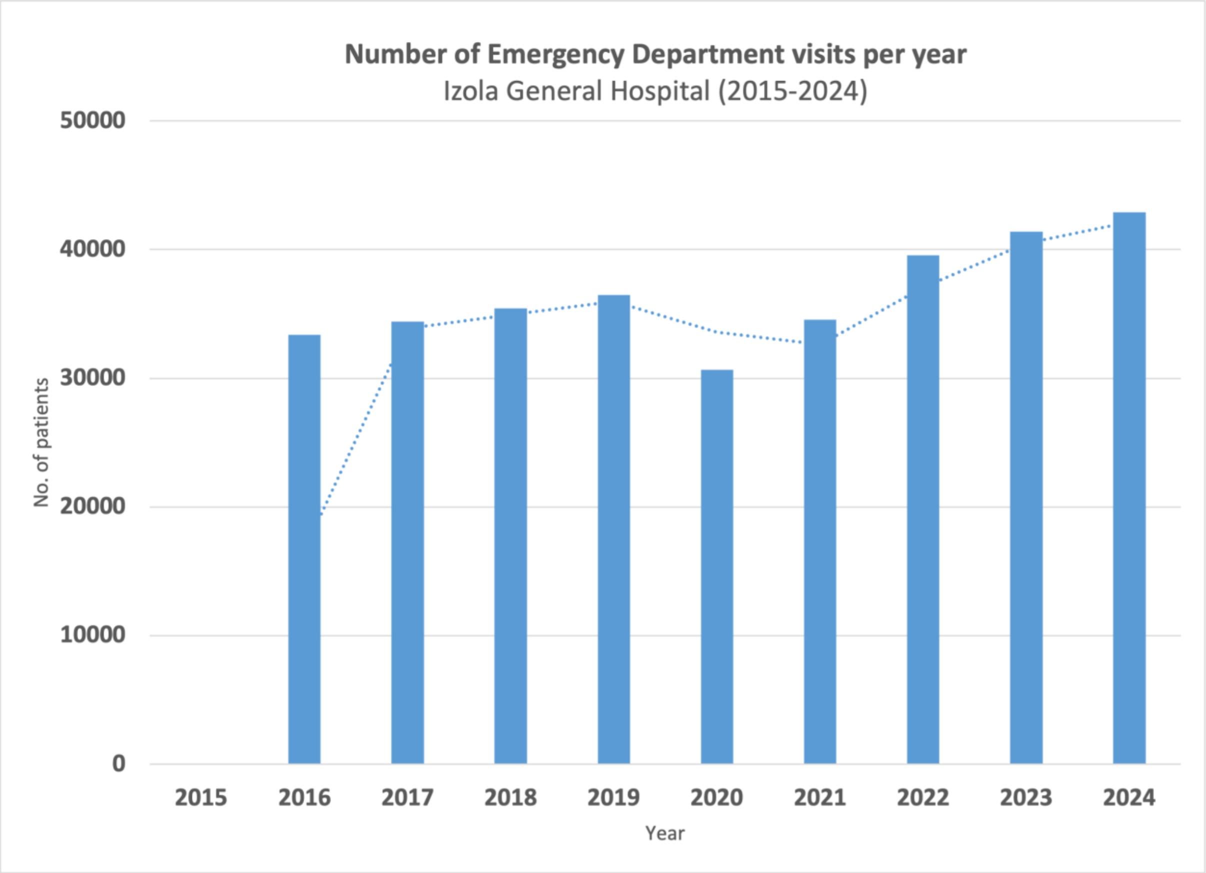

Supplementary Figure S1. Number of Emergency department visits during the study period. The opening of the department dates to the begining of 2026.
